# IgA nephropathy in Salvador, Brazil: a more aggressive disease?

**DOI:** 10.1590/2175-8239-JBN-2018-00030002

**Published:** 2018-09-21

**Authors:** Rui Toledo Barros

**Affiliations:** 1 Universidade de São Paulo, Faculdade de Medicina, São Paulo, SP, Brasil.

The immunoglobulin A nephropathy (IgAN) was first described in France by Berger and Higlais in 1968. Since then, studies worldwide have proved it to be the most common glomerulonephritis in the world.^1^ IgA nephropathy is defined by the predominant or co-dominant IgA-containing immune complex deposits in the kidney. As such, its diagnosis requires the pathologic evaluation of invasive renal biopsies by light- and immunofluorescence microscopy. It was initially thought of as a benign disease, as it is evident from its earlier synonym of "benign hematuria" and confined in France. However, subsequent long-term follow-up studies worldwide have proved both the above assumptions wrong, because this disease has variable outcomes.^1^^,^ 2

Epidemiological studies from different parts of the world have proven that IgAN has global distribution, and it is the most common primary glomerular disease. There is however, significant variability in the reported incidences and prevalences in different countries. High rates of 20 to 47 percent have been reported in biopsy studies from Western Europe, parts of Asia and Australia. On the other hand, very low rates have been reported from the United States, Africa, Middle East and some parts of Asia.^3^ The apparent variable rates most probably reflect differences in the ethnical constitution of the population, medical practices, biopsy policies and lack of IF facilities in some countries. In Brazil, data concerning the prevalence and impact of IgAN is scarce. A recent paper involving 9,617 kidney biopsies in the country depicted 20.1% prevalence of IgAN among primary glomerulopathies.^4^ The Paulista Registry of Glomerulonephritis reported the prevalence of 17.8%^5^ and IgAN accounts for varying ranges of 2-25% of primary glomerulonephritis, according to other local reports.^5^^,^ 6

Although this disease was initially considered benign, it is now known to lead to a slowly but progressive decline in renal function and end-stage renal disease, developing in up to 30% of patients 20 years after the diagnosis.^1^^,^ 2 Long-term outcome data shows variable rates of disease progression throughout the world. Attempts have been made to identify clinical, laboratory and morphologic features in renal biopsies, which can predict outcome.

In the paper published in this issue of the *Brazilian Journal of Nephrology*, Souza *et al.* analyzed the clinical and histological patterns of 32 patients with IgAN in Salvador, Brazil^7^. The prevalence of IgAN was 6% in the primary glomerulonephritis cases; and chronic histological lesions and poor laboratory markers were frequent. Significant proteinuria (> 2.0 g/24 hours) and hypertension were found in most patients and the data suggests poor chronic kidney disease outcome.

Despite the limitations of a retrospective study, and its small sample size, it adds relevant information regarding histological analyses according to the Oxford classification of IgAN.^8^^,^ 9 A high frequency of scores associated with progressive kidney disease was reported in this cohort: segmental sclerosis in 81% and tubular-interstitial lesions in 44% of patients.

Overall, the above findings hint at some questions: are there any genetic predispositions/polymorphisms in this study population which affect the presence of a more aggressive disease? Is the high frequency of scores associated with chronic lesions only related to delayed referral to medical resources and delayed indications of renal biopsies? These questions, therefore, call for early intervention strategies as well screening programs, not only to identify and treat the patients, but also for a better understanding of the factors which lead to the progression of this disease.
